# Unified syntax in the bilingual mind

**DOI:** 10.3758/s13423-019-01666-x

**Published:** 2019-12-10

**Authors:** Mathieu Declerck, Yun Wen, Joshua Snell, Gabriela Meade, Jonathan Grainger

**Affiliations:** 1 Laboratoire de Psychologie Cognitive, Aix-Marseille University and Centre National de la Recherche Scientifique, Centre St. Charles, 3 place Victor Hugo, 13331 Marseille, France; 2 Joint Doctoral Program in Language and Communicative Disorders, San Diego State University and University of California, San Diego, San Diego, CA USA; 3 Institute for Language Communication and the Brain, Aix-Marseille University, Marseille, France

**Keywords:** Bilingualism, Syntax, Language comprehension, Parallel word processing

## Abstract

Are syntactic representations shared across languages, and how might that inform the nature of syntactic computations? To investigate these issues, we presented French-English bilinguals with mixed-language word sequences for 200 ms and asked them to report the identity of one word at a post-cued location. The words either formed an interpretable grammatical sequence via shared syntax (e.g., *ses feet sont big* – where the French words *ses* and *sont* translate into *his* and *are,* respectively) or an ungrammatical sequence with the same words (e.g., *sont feet ses big*). Word identification was significantly greater in the grammatical sequences – a bilingual sentence superiority effect. These results not only provide support for shared syntax, but also reveal a fascinating ability of bilinguals to simultaneously connect words from their two languages through these shared syntactic representations.

## Introduction

More than half a century of research on the processing of linguistic information by bilinguals has addressed two main questions: To what extent can processing proceed in parallel in two languages and, when possible, are the representations involved in this processing shared by the two languages? In the current study, we focused on the latter issue and, more specifically, on the hypothesis that bilingual language comprehension involves language-independent syntactic representations.

While several studies have investigated whether syntactic structures are shared across languages in bilinguals, these studies have almost exclusively examined language production using a cross-linguistic version of the syntactic priming paradigm (for a recent review, see van Gompel & Arai, 2018). In the seminal study of Hartsuiker, Pickering, and Veltkamp (2004), Spanish-English bilinguals more often used a passive sentence to describe a picture in their second language (L2) if they had previously processed a passive sentence in their first language (L1), relative to when they had previously processed an active sentence in their L1. This finding was taken as clear evidence in favor of the shared syntax hypothesis, the general idea being that residual activation of the syntactic structures used in producing in language A can only influence subsequent processing in language B if the same language-independent structures are at play. Since then, cross-language syntactic priming effects have been obtained with many different language combinations and sentence structure types (e.g., Huang, Pickering, Chen, Cai, Wang, & Branigan, 2019; Jacob, Katsika, Family, & Allen, 2017; Shin & Christianson, 2012).

Given the evidence for shared syntax in bilingual language production, it seems logical to hypothesize that bilingual language comprehension also involves language-independent syntactic representations (for a review on the syntactic overlap between production and comprehension, see Indefrey, 2018). However, the paradigm of choice for revealing shared syntax in language production – syntactic priming – has yielded highly elusive evidence for such effects, even in monolingual language comprehension (for a review, see Tooley & Traxler, 2010; however, see e.g., Giavazzi, Sambin, de Diego-Balaguer, Le Stanc, Bachoud-Lévi, & Jacquemot, 2018; Tooley, Pickering, & Traxler, 2019). The limited data addressing this issue in a purely bilingual language comprehension context are also inconclusive. For example, Weber and Indefrey (2008) failed to find cross-language syntactic priming in bilingual language comprehension, but they did find evidence in line with the shared syntax hypothesis in a later study (Weber & Indefrey, 2009; see also Kidd, Tennant, & Nitschke, 2015).

In the current study, we moved away from overt syntactic priming manipulations in an attempt to provide a more direct measure of how shared syntax might influence sentence comprehension processes in bilinguals. To do this, we capitalized on the recently reported sentence superiority effect observed with the Rapid Parallel Visual Presentation (RPVP) paradigm (Snell & Grainger, 2017; Wen, Snell, & Grainger, 2019). The sentence superiority effect entails more accurate word identification in a grammatically correct sequence (e.g., *our fox can fly*) than in a scrambled ungrammatical sequence (e.g., *our can fly fox*). In Snell and Grainger (2017) and Wen et al. (2019), French participants were briefly presented with a string of four French words. This string of words could either be a grammatically correct sequence or an ungrammatical sequence of the same words. After 200 ms, the four words were replaced by hash masks and a cue appeared above one of the four masks. The task was to identify the word that had been presented at the cued location. Results showed that identification of the cued word was more accurate when the target word was part of a grammatically correct sequence than when it was part of an ungrammatical sequence. These results point to a system that can very rapidly generate a sentence-level representation on the basis of partial information about word identities and their parts-of-speech (e.g., noun, verb, or adjective). Sentence-level syntactic representations then constrain the identity of a word at a given location by specifying the probability that a given part-of-speech is present at that location (see Snell, Meeter, & Grainger, 2017, for additional evidence and a model of parallel syntactic processing).

In the present study, we implemented a bilingual version of the RPVP paradigm by intermixing words from two languages. French-English bilinguals were presented with two French and two English words intermingled within either a grammatically correct sequence or an ungrammatical sequence generated by shuffling word order. The grammatically correct sequences were correct in the sense that translating two of the words to produce a monolingual sequence always resulted in a well-formed grammatical sequence in either language. We reasoned that observing a sentence superiority effect in these conditions would provide strong support for the shared syntax hypothesis. A language-specific syntax hypothesis, on the other hand, predicts no sentence superiority effect with mixed-language sequences, since any attempt to generate a syntactic representation on the basis of language-specific syntactic representations should fail in these conditions. Additionally, the types of errors that participants produce may be informative as to whether syntax is shared across languages. If syntactic representations constrain word identification by specifying a language-independent part-of-speech at each position in the sequence, then errors should more likely have the same part-of-speech as the target word. These syntactic cues should only influence processing in well-formed grammatical sequences.

## Method

### Participants

Data from 24 native French speakers who were highly proficient in English are reported (13 female, mean age = 24.2 years).^1^ To qualify for this study, the French-English participants had to obtain a score of 80% or higher (average score: 85.7%) on an English vocabulary test (i.e., lexical decision task of 60 written items; Lemhöfer & Broersma, 2012), indicating that they are highly proficient in English. Prior to the experiment, the French-English bilinguals filled in a language history questionnaire (see Table 1). Data from an additional two participants were excluded due to a technical issue.

**Table 1:** Overview of demographic information

	French	English
Age of acquisition	0.8 (1.5)	9.0 (5.0)
Currently used	78.5% (14.0)	21.5% (14.0)
Speaking	6.7 (0.4)	5.3 (0.9)
Writing	6.2 (0.9)	5.0 (1.0)
Reading	6.6 (0.7)	5.5 (1.0)

The information consists of the average age of acquisition of each language and the average percentage of time the participants currently used each language. Additionally, the average self-rated scores for speaking, writing, and reading each language are given, ranging from 1 (very bad) to 7 (very good). Standard deviations are presented in brackets

### Stimuli

Two hundred low semantically constraining sequences were constructed that were grammatically correct in both French and English. Each of these sequences consisted of four words (between two and six letters long) that had orthographically distinct translation equivalents (i.e., non-cognates). Two of the words were in French and the other two words were in English. The position of French and English words was random within each sequence (e.g., *ses feet sont big* [English: his feet are big], *play with une amie* [English: play with a friend], and *elle comes with nous* [English: she comes with us]), so that the participant could not predict the language of all words based on the language of one word.

For each grammatically correct sequence, an ungrammatical sequence was constructed that was grammatically incorrect in both languages (e.g., grammatically correct: *ses feet sont big* vs. ungrammatical: *sont feet ses big*). Each participant saw either the grammatically correct sequence or the corresponding ungrammatical sequence (counterbalanced across participants).

Each sequence had one word marked that had to be typed after the string of words was masked. This target word occurred in the same position relative to the other words for the respective grammatically correct and ungrammatical sequences. An equal number of target words were used in each of the four word positions. Furthermore, about half of the target words were French (101 out of 200) and the others were English (99 out of 200).

### Procedure

The study was approved by the Comité de Protection des Personnes SUD-EST IV (No. 17/051). All participants gave their written informed consent before the experiment started. Prior to the experiment, both oral and visual instructions were provided about the task and eight practice trials were presented. The main experiment consisted of 200 trials.

**Fig. 1:**
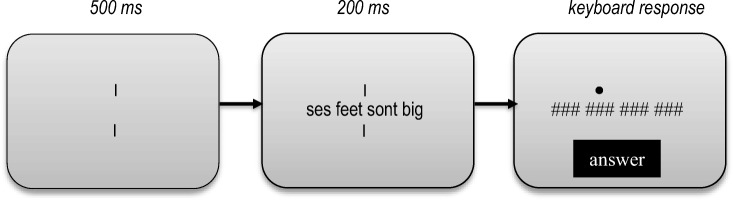
Illustration of a trial sequence of the bilingual Rapid Parallel Visual Presentation procedure. In the example here, the target word (“feet”) is embedded in a grammatically correct sequence. An example of a corresponding ungrammatical sequence would be “sont feet ses big” with the target word at the same position

The procedure of this study was identical to that of Snell and Grainger (2017). More specifically, each trial started with two vertical bars in the middle of the screen (see Fig. 1 for an example of the trial sequence). After 500 ms, four words appeared between these two bars, which stayed on the screen for 200 ms, after which each letter was replaced by a single hash mark. At the same time as the hash mark mask, a cue was presented above the target word that had to be identified. This configuration remained on the screen until the participant had finished typing a word – which could be seen under the hash marks – and pressed the return key. Finally, feedback was given for 600 ms in the form of a green or red dot for correct or incorrect responses, respectively.

### Analysis

All responses that differed from the target word in any way were considered errors. For the purposes of the part-of-speech error analysis, the following error trials were excluded: trials with no response, nonwords, and words with an ambiguous part-of-speech relative to the target word. In total, 901 error trials were categorized as having the same or a different part-of-speech as the target word.

The data were analyzed using logistic mixed-effects regression modeling (Jaeger, 2008). Both participants and items were considered random factors with the fixed effect (grammatical context) varying by all random factors (Barr, Levy, Scheepers, & Tily, 2013). Finally, *z*-values larger or equal to 1.96 were deemed significant (Baayen, 2008).

## Results

The shared syntax hypothesis was confirmed, as significantly more words were identified correctly in grammatically correct sequences (70.3%) than in ungrammatical sequences (63.0%), *b* = 0.50, SD = 0.12, *z* = 4.12. To examine the influence of the language of the target word, we also ran an analysis with both grammatical context and target language as independent variables.^2^ The results showed that the bilingual sentence superiority effect was similar when the response language was French (6.0%) or English (8.8%), *b* = 0.21, SD = 0.15, *z* = 1.39.

**Fig. 2:**
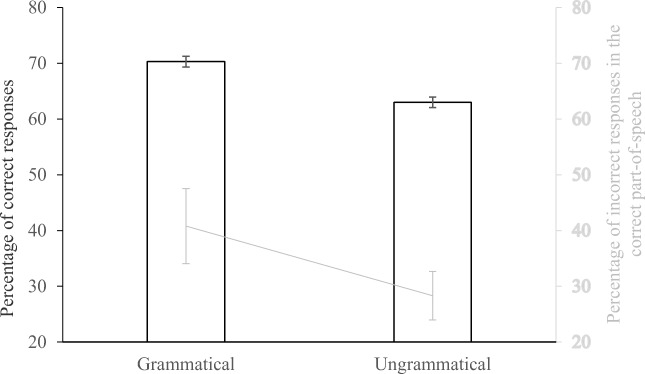
The black bars indicate the mean percentage of correct responses for grammatically correct and ungrammatical sequences (left axis). The gray line indicates the mean percentage of errors that had the same part-of-speech as the target word for grammatically correct and ungrammatical sequences (right axis). Error bars indicate 95% confidence intervals (Cousineau 2005)

We investigated the error trials further to determine whether or not the incorrect response had the same part-of-speech as the intended target word. The results of this analysis showed that incorrect word responses belonged to the same part-of-speech as the target word more often in grammatically correct sequences (40.8%) than in ungrammatical sequences (28.3%), *b* = 0.88, SD = 0.28, *z* = 3.16 (see Fig. 2).

## Discussion

The goal of the present study was to investigate whether syntax is shared across languages in bilinguals. To this end, we presented sequences of four words to French-English bilinguals and asked them to identify one of the words at a post-cued location. The word sequences were composed of two words from each language and could either form a grammatically correct sequence or an ungrammatical re-ordering of the same words. Word identification was found to be more accurate in the grammatically correct sequences than in the ungrammatical sequences – a bilingual sentence superiority effect. Furthermore, we found that incorrect word responses were more likely to have the same part-of-speech as the target word in the grammatically correct context compared to the ungrammatical context.

This bilingual sentence superiority effect is unequivocal evidence in support of the shared syntax hypothesis (Hartsuiker et al., 2004). Prior evidence for shared syntax in bilinguals had mainly been reported in the form of cross-language syntactic priming during language production. In such situations, the inference for shared syntax is based on the lingering influence of the syntactic structure involved in processing in one language on the syntax used in processing the other language in a subsequent trial. Additional evidence for shared syntax comes from the fact that code-switching is a common practice in certain bilingual communities (e.g., Myers-Scotton, 1993). The present findings provide even further compelling evidence for shared syntax by demonstrating that words in the two languages can simultaneously contribute to the generation of a common syntactic structure.

One might argue that the bilingual sentence superiority effect was observed because the participants translated, for example, all L2 words swiftly into L1 words. Consequently, the grammatically correct sequences could be processed as valid grammatically correct L1 sequences. If this were the case, then a large proportion of incorrect responses should be translation-equivalents of the correct responses. Yet, only 1.7% (i.e., 27 responses across all participants) of all incorrect responses were translation-equivalent words. So, it seems unlikely that the participants translated all words into one language to process the sequences in a homogenous language context.

Because half of the words are in English and the other half in French, some of the sentences have adjacent words in the same language. It could be that these same-language word pairs caused the apparent bilingual sentence superiority effect in the current study. To examine whether this was the case, we reanalyzed the target words on the outer positions of the four-word sequence (i.e., word positions one and four in a four-word sequence), since the adjacent word could either be from the same language or from the other language, whereas the target words from inner positions could have both.^3^ The results showed that an effect of grammatical context was still present when the adjacent word was from the other language (grammatical: 61.5% correct; ungrammatical: 50.6% correct), *b* = 0.75, SD = 0.21, *z* = 3.64. Moreover, the interaction between the language of the adjacent word (same vs. different) and grammatical context was not significant, *b* = 0.43, SD = 0.27, *z* = 1.63. As a matter of fact, the averages indicated that the grammatical context effect was even larger when the adjacent word was from the other language (10.9% vs. 4.5%). Hence, it is clear that our sentence superiority effect was not being driven by cases where two adjacent words were from the same language.

In order to account for the present findings, we assume that our participants rapidly generated a sentence-level representation on the basis of partial information about word identities and their parts-of-speech. The sentence-level representation then constrains the identity of a word at a given location by specifying the probability that a given part-of-speech is present at that location (Snell et al., 2017). We assume that syntactic parses are initiated on the basis of information about the parts-of-speech of the words in a sequence and the ordering of these words. In line with Harstuiker et al. (2004), we further assume that parts-of-speech are language independent, such that the same representation can be used to indicate that *tree* is a noun and that *arbre* (meaning *tree* in French) is a noun, for example. Rapidly identifying the parts-of-speech for some of the words in a sentence provides a means to initiate a tentative parse and to generate probabilities for the parts-of-speech of the other words in the sentence. Thus, for example, given the sequence “*ses feet sont big,*” knowing there is a noun at position 2 and a verb at position 3 provides strong constraints on the type of word that can occur at positions 1 and 4 in a grammatically correct sequence. The precise nature of this rapidly generated syntactic parse remains to be investigated in future work.

What might be the nature of this rapidly generated language-independent syntactic parse? Although a complete answer to this question is beyond the scope of the present work, we suggest that one of its key characteristics must be its preliminary, approximate nature, constructed on the basis of limited evidence extracted in parallel from several words (cf. Snell et al., 2017; and see Wen et al., 2019, for evidence concerning the timing of the sentence superiority effect based on electrophysiological data). Whatever the precise nature of this preliminary parse, as information continues to accrue, the parse will either be confirmed and completed, or replaced by an alternative parse. Here, we note for comparison the evidence in favor of “good-enough” syntactic representations in language comprehension (Ferreira & Lowder, 2016), as well as the notion of a “skeleton parse” used in corpus linguistics (Leech & Garside, 1991).

Finally, the present results have repercussions that go beyond the shared syntax hypothesis. Our findings provide additional evidence for language non-selective processing during bilingual language comprehension, given that word representations from both languages must be processed simultaneously in order to account for our findings. Prior support for the non-selective access hypothesis (e.g., Grainger & Dijkstra, 1992) was obtained by revealing an influence of lexical representations in the non-target language during processing of a word in the target language (e.g., Thierry & Wu, 2007; van Heuven, Dijkstra, & Grainger, 1998; however, see Costa, Pannunzi, Deco, & Pickering, 2017). We have demonstrated that words from both languages are not only activated but can also jointly participate in the construction of a syntactic representation. This implies that any top-down control over lexical activity in one or the other language was deactivated (or at least diminished) in the present experiment, most probably because there was no single target language.

In sum, our findings revealed the outstanding ability of bilinguals to simultaneously process syntactic information from words in two languages, and to use the tentative parse generated on the basis of this information to constrain the on-going processing of word identities in both languages. These findings not only provide strong support for parallel language processing, but also crucially demonstrate that syntactic representations can be shared across languages in bilinguals. Moreover, they provide further support for the hypothesized parallel processing of syntactic information across multiple words during reading (Snell et al., 2017), this time in the form of a bilingual sentence superiority effect.

### Open practices statement

The data and materials are available on the Open Science Framework (https://osf.io/vnwfb/).

## Footnotes

1 A power analysis for data with random participants and items was used to determine the number of participants (Green & Mcleod, 2016). More specifically, 200 (Monte Carlo) simulations, as suggested by Brysbaert and Stevens (2018), were run using the simr package in R on the data of Wen et al. (2019). The results showed that with 24 participants, power was 100% to observe the main effect that the current study focuses on. Since this data is based on single language processing, and the current study relies on mixed language processing, which can decrease performance (for a review, see Declerck & Philipp, 2015), we kept 24 participants to have an optimal chance of observing the effect of interest.

2 A convergence issue occurred for the analysis that included target language. To overcome this issue, we determined the maximal random effects structure permitted by the data (cf. Barr et al., 2013), which led to a model with random intercepts and random by-participant slopes for target language and grammatical context.

3 We also investigated whether the grammatical context effect differed for target words appearing at the two outer (words at positions 2 or 3) vs. two inner (words at positions 1 or 4) positions. The results showed that there was no difference for grammatical context between these positions, *b* = 0.07, SD = 0.15, *z* = 0.43.
